# Quality of Life and Mental Health in Kidney Transplant Recipients During the COVID-19 Pandemic

**DOI:** 10.3389/fpsyt.2021.645549

**Published:** 2021-06-09

**Authors:** Concetta De Pasquale, Maria Luisa Pistorio, Pierfrancesco Veroux, Rossella Gioco, Alessia Giaquinta, Francesca Privitera, Massimiliano Veroux

**Affiliations:** ^1^Vascular Surgery and Organ Transplant Unit, Department of Educational Science, University of Catania, Catania, Italy; ^2^Vascular Surgery and Organ Transplant Unit, Department of General Surgery and Medical-Surgical Specialties, University Hospital of Catania, Catania, Italy; ^3^Organ Transplant Unit, Department of Surgical and Medical Sciences and Advanced Technologies, University Hospital of Catania, Catania, Italy; ^4^Endocrine Surgery Unit, Department of Surgical and Medical Sciences and Advanced Technologies, University Hospital of Catania, Catania, Italy

**Keywords:** COVID-19, quality of life, psychopathology, kidney transplantation, mental health

## Abstract

**Introduction:** The COVID-19 pandemic has led to an increase in mental distress such as phobic anxieties, depressive reactions, hypochondriac concerns, and insomnia. Among the causes are risk of infection and prolonged isolation. This study aimed to analyze psychopathological variables and dysfunctional lifestyles related to adequate therapeutic compliance in kidney transplant recipients.

**Methods:** Eighty-nine kidney transplant recipients were evaluated using an online protocol including a questionnaire concerning habits, lifestyle and psychophysical well-being in the COVID-19 period, the Middlesex Hospital Questionnaire (MHQ) and the SF-36 Health Survey to evaluate the perception of their physical and emotional health.

**Results:** Of these recipients, 28.6% reported changes in their emotional state. Sleep quality deteriorated for 16.1%. Anxiety (M = 5.57, *r* = 0.33; *p* < 0.05) and phobia (M = 6.28, *r* = 0.26; *p* < 0.05) correlated with concerns related to physical health. There was no negative impact on relational and socialization aspects, which were likely well compensated by the use of remote technologies such as video phone calls, Zoom meetings and use of computers (*r* = 0.99; *r* = 0.80; *p* < 0.05).

**Conclusions:** It would be interesting to maintain this remote visit and interview mode to monitor, on a clinical and psychological level, kidney transplant recipients in subsequent follow-ups (12–18 months), to check for any psychopathological disorders and/or changes in their resilience capacity in the Coronavirus emergency.

## Introduction

Coronavirus disease 2019 (COVID-19), which began in December 2019, and the consequent containment measures (quarantine or isolation) applied in Italy in March 2020, has had an influence on mental health (anxiety symptoms, depression), as already evidenced by some studies carried out in China (1–3). Zhang and Ma showed a dissatisfaction with life in subjects in isolation, more marked in those subjects who were previously very active and found themselves forced into a sedentary life, generating feelings of boredom, dissatisfaction, frustration and anger (4). The stress and consequences associated with isolation linked to COVID-19 have also led to disturbances in the biological sleep-wake rhythm (5). The causes of stress have been numerous: fear of contagion, prolonged isolation, denied grieving, and fears for hospitalized relatives for whom visits are not allowed with inevitable effects on mental health (5, 6). Following isolation, another interesting fact reported in the literature concerns the loss of contact with external events, the mass media becoming the only source of information, but the topic dealt with refers only to the epidemic with some of the information being incorrect or contradictory (7–9). According to Steven Stosny, the spasmodic search for information can cause “headline stress disorder,” an anxiety disorder linked to the media, characterized by a feeling of stress and anxiety (10). Another psychopathological consequence of the pandemic and isolation linked to the risk of infection is depressive disorder. While measures of social isolation and distancing are synonymous with protection for oneself and for others, they can also lead to a feeling of helplessness, mistrust and exclusion. A Chinese study, in the 2 months following the outbreak of the COVID-19 pandemic, reported a 20.1% rate of depression in 7,236 healthy volunteers (11). Regarding the perception of the risk of contagion and the level of stress caused by COVID-19, the problem becomes more evident for subjects with chronic diseases such as cancer patients and immunosuppressed patients (including transplant recipients) as chronic immunosuppression is a known risk factor for viral and bacterial infections. However, it is also essential to counteract the uncontrolled antiviral inflammatory response and prevent transplant rejection (12–14).

Transplant recipients receiving immunosuppressive therapy are at the highest risk of severe illness and therefore they are high-risk patients for the negative outcome from COVID-19 (15).

One of the particular aspects of the life of the transplant recipient is that, in the post-operative phase, the patient lives in a condition of “isolation,” having to pay particular attention to their “living” environment and preferring a limited social life since immunosuppressive treatment involves immunosuppression in the patient. As shown in different studies, these particular conditions, in kidney transplant recipients can materialize in mental disorders such as post-traumatic stress disorder, adaption disorder, and psychosomatic disorder (16). With COVID-19, as in a post-operative situation, social isolation has been implemented.

Based on a possible negative impact of COVID-19 and isolation on recipients' psychological health, the psychiatric and psychological team of the transplant center of the University hospital of Catania, Italy, continued with their meetings and follow-ups of kidney transplant recipients online; this was due to the closure of the clinics. Positive responses from patients receiving such “care” encouraged the data collection for the present study.

The aim of our research was to explore the impact of COVID-19 and the consequent period of isolation on the perception of health in general, on therapeutic adherence and on emotional states of kidney transplant recipients, using a questionnaire built *ad hoc* by the authors. In addition, the study aimed to assess quality of life (compared to the previous year) and main psychic symptoms and relevant traits in the recipient during the COVID-19 pandemic, using standardized tests.

## Methods

### Study Participants

During isolation and major restrictive measures from COVID-19, 110 kidney transplant recipients of both sexes aged 23–75 years with an education level of ≥12th grade (high school diploma or higher) were evaluated at attending the Organ Transplant Unit, University Hospital of Catania, Italy. In the period between March 15, 2020 and June 15, 2020. They were contacted by telephone in order to monitor their psycho-physical well-being, therapeutic adherence and guarantee psychological support.

After the first telephone contact, appointments were established every 2 weeks in which interviews were carried out online by video calls, during which the patients expressed their desire to share their experiences of living through such a difficult period. Given the positive feedback from patients at the follow-ups performed online, it was decided to ask them for their participation in this study and to compare their self-perceived general health status compared to the previous year. The SF-36 data from the previous year had been obtained by us during the normal psychological follow-up expected for all kidney transplant recipients.

The 110 patients who during the indicated period should have had on-site checks at the Transplant Center to monitor renal function were contacted. They were asked to participate in the study that involved completing an online questionnaire on psychophysical well-being during the COVID-19 pandemic. Twenty-one recipients refused to participate. The questionnaire was emailed to 89 kidney transplant recipients. We asked patients to fill in the questionnaire independently, avoiding the help of family members. All recipients included in the study were receiving standardized immunosuppressive therapy with tacrolimus, mycophenolate mofetil, and steroids; none of them was taking psychiatric drugs. All transplant recipients provided their informed consent to participate in the study. Informed consent was sent by email and participants printed, signed, scanned and sent it back by email. The local ethical committee approved the study procedures in accordance with the Ethical Principles for Medical Research Involving Human Subjects indicated by the 2004 World Medical Association Declaration of Helsinki (17).

### Instruments

An online questionnaire was developed as a consequence of the interruption of post-transplant follow-up checks in the outpatient clinics of our Transplant Center. The online protocol included a questionnaire, built *ad hoc*, concerning the emotional state and psychophysical well-being in the period of COVID-19, consisting of 8 questions. Recipients were asked to rate: (1) Due to the COVID-19 pandemic how is your overall health? (rated on a 5-point Likert scale: 0 = Excellent, 4 = poor); (2) Has the idea of staying at home, due to COVID-19 isolation, changed your emotional state? (rated on a 5-point Likert scale: 0 = not at all, 4 = very much); (3) If so, how did your emotional state change? How do you feel? (multiple choices possible; such as “I feel worried and anxious”); (4) How worried about your health are you during the COVID-19 pandemic? (rated on a 5-point Likert scale: 0 = Much less than usual, 4 = Much more than usual); (5) Has your sleep quality deteriorated in the last few weeks? (rated on a 5-point Likert scale: 0 = not at all, 4 = very much); (6) In this period of possible COVID-19 contagion, are you afraid to go to the hospital for periodic outpatient checks? (rated on a 5-point Likert scale: 0 = Never, 4 = always); (7) Can you take immunosuppressants in the same way as before COVID-19, according to the doctor's recommendations? (0 = yes, 1 = no); (8) If you were unable to take immunosuppressive therapy in the same way as before COVID-19, why? (free answer). A total score was calculated (maximum total score = 24), which expresses the “burden related to the pandemic from COVID-19”: the higher the score, the higher the burden caused by COVID-19 (scale reliability: Cronbach's alpha = 0.71). Questions n. 3 and n. 8 are not included in the calculation of the total score, as question n. 3 provides a multiple-choice answer from a series of statements, and question n. 8 provides a free answer.

In addition to the specific questions about COVID-19, the online questionnaire included The Middlesex Hospital Questionnaire (MHQ), which provides a simple and rapid quantification of the main symptoms and relevant traits: fluctuating anxiety (ANX), phobic anxiety (PHOB), obsessive-compulsive traits (OBS), somatic symptoms (SOM), depression (DEP), and hysteria (HY) (18). It is made up of 48 items, partly dichotomous (no/yes−0/2), partly assessed on a three-level scale (0–1–2) of frequency or severity of the symptom or behavior explored. The normal reference values, for each of the traits evaluated, are the following: 5.1 (ANX), 2.9 (PHOB), 5.8 (OBS), 3.2 (SOM), 3.3 (DEP), 7.5 (HY); the SF-36 was used to assess the perception of physical and emotional health (19). The Short Form (36) Health Survey is a 36-item, patient-reported survey of patient health. The SF-36 consists of eight scaled scores, which are the weighted sums of the questions in their section. Each scale is directly transformed into a 0–100 scale on the assumption that each question carries equal weight. The lower the score the more disability. The higher the score the less disability. The eight sections are: vitality (VT), physical functioning (PF), bodily pain (BP), general health perceptions (GH), physical role functioning (PR), emotional role functioning (ER), social role functioning (SR), and mental health (MH).

The complete questionnaire was sent by email via a reference link. The information was collected in an archive (google modules) and automatically linked to a spreadsheet (20).

In addition, the authors decided to evaluate any differences in the mental health index (MHI) and physical health index (PHI) measured with the SF-36 during the pandemic, compared to the previous year. This was made possible because the sample of recipients participating in the study had already been assessed, through the SF-36, during periodic post-transplant psychological follow-ups, but prior to the pandemic.

### Statistical Analysis

Statistical analyses were conducted using SPSS 24. The Kolmogorov–Smirnov test ascertained normal distribution of data. The Pearson correlation coefficient r was used for the analysis between Burden COVID-19, some demographic characteristics of the sample, MHQ and SF-36. In addition, we applied a multivariate linear regression analysis to predict values of outcome variables (symptoms of MHQ) from predictor variables (Burden COVID-19).

Moreover, we compared, by means of *t*-test for paired samples, the mental health index (MHI) and the physical health index (PHI) of the SF-36 1 year before the COVID-19 emergency (March–June 2019) and after 1 year, coinciding with the pandemic and the lockdown period.

## Results

Demographic characteristics of patients included in the study are shown in Table 1.

**Table 1:** Demographic characteristics of patients included in the study.

Age, mean ± SD (range)	52.48 ± 13
Male sex	42.64%
**Donor source**	
Living donor	21.54%
Deceased donor	78.46%
BMI (Kg/m^2^)	31.2 ± 2.3
**Education**	
High school diploma or degree	76.48%
Middle school or lower	23.52%
**Length prior dialysis treatment**	
More than 3 years	24.87%
One to 3 year	75.13%
Serum Creatinine (mg/dL)	1.64 ± 0.92
mGFR (mL/min/1.73 m^2^)	65.42 ± 22.53
Time post-transplant (years)	6.71 ± 4.37
**Original Nephrologic disease**	
Hypertensive nephrosclerosis	19%
Diabetic nephropathy	9%
Chronic glomerulonephritis	40.87%
Polycystic kidney	24.43%
Unknown	6.7%

### General Health Before and After COVID-19

From the answers to the questionnaire concerning the emotional state and psychophysical well-being in the period COVID-19, completed by 89 kidney transplant recipients, in 50% (45) of the sample, the COVID-19 pandemic did not change the perception of their general state of health prior to the COVID-19 period (Item n. 1), nor was any concern about illness recorded. Specifically, 48.3% (43) of transplant recipients reported having “good” general health and 12.1% (11) reported “passable.”

### Health Concerns and Emotional Experience Due to Isolation

Regarding concerns for their health (Item n. 4), 37.9% (34) reported that they were worried “more than usual,” and 12.1% (11) “much more than usual.” With regard to their emotional experience, due to the isolation put in place by the Italian Government (Item n. 2), 27.6% (25) of recipients declared that they had changed their emotional state “enough,” and 10.3% (9) “a lot.” Regarding their emotional state (multiple choice answer of Item n. 3), 41.4% (37) of subjects reported anxiety, and 17.2% (15) reported low energy and apathy.

### Sleep Disturbances and Fear of Contagion

Concerning the sleep-wake rhythm (Item n. 5), 15.5% (13) of recipients reported a worsening of their sleep quality. Regarding the question relating to the fear of going to the reference Transplant Center for their periodic follow-up checks (Item n. 6), 34.8% (30) replied “sometimes,” and 30.4% (27) “often.”

### Impact of COVID-19 on Therapeutic Adherence

Finally, the COVID-19 pandemic and the period of isolation does not seem to have had any influence on therapeutic adherence to immunosuppressive therapy (Item n. 7 and Item n. 8), which seems to have remained adequate for almost all of the sample (93.1%) (83).

Correlations (Pearson *r*) between the demographic variables of the sample, the burden of COVID-19, the SF-36, and the MHQ are shown in Table 2. There were significant positive correlations between age (Age/Burden C-19 *r* = 0.830 *p* < 0.05), level of education (E/Burden C-19 *r* = 0.441 *p* < 0.05), time spent on dialysis (TD/Burden C-19 *r* = 0.532 *p* < 0.05), years since transplantation (YT/Burden C-19 *r* = 0.763 *p* < 0.05) and Burden of COVID-19 (Burden C-19). Regarding the main symptoms and relevant traits measured with the MHQ, Obsessions (OBS/Burden C-19 *r* = 0.180 *p* < 0.05) and Hysteria (HY/Burden C-19 *r* = 0.361 *p* < 0.05) dimensions were positively correlated with the Burden of COVID-19. A positive correlation also emerged between age and Obsessions (Age/OBS *r* = 0.547 *p* < 0.05), level of education and somatizations (E/SOM *r* = 0.481 *p* < 0.05). Furthermore, the higher their level of education, the better their mental health, measured with the SF-36 (E/MH *r* = 0.990 *p* < 0.05).

**Table 2:** Correlations through pearson coefficient (r) between MHQ, SF-36, Burden C-19, and demographic characteristics of patients included in the study.

	**ANX**	***DEP***	***SOM***	***PHOB***	***OBS***	***HY***	***PF***	***PR***	***BP***	***GH***	***BT***	***SR***	***ER***	***MH***	***Burden C-19***
Sex	0.213	0.832^*^	0.029	0.001	0.303	0.499	0.100	0.125	0.806	0.948^*^	0.006	0.448	0.053	0.006	
Age	0.114	0.585^*^	0.293	0.321	0.547^*^	0.108	0.001	0.449	0.091	0.581	0.914	0.000	0.722	0.952	0.830^*^
E	0.034	0.264	0.481^*^	0.323	0.234	0.012	0.000	0.718	0.087	0.281	0.943	0.110	0.875	0.990^*^	0.441^*^
TD	0.443^*^	0.980^*^	0.469^*^	0.714^*^	0.859^*^	0.502^*^	0.252	0.270	0.376	0.079	0.414	0.347	0.298	0.882	0.532^*^
YT	0.047	0.316^*^	0.448^*^	0.160	0.657^*^	0.399^*^	0.672	0.304	0.519	0.647^*^	0.378	0.713^*^	0.299	0.459^*^	0.763^*^
Burden C-19	0.013	0.006	0.000	0.046	0.180^*^	0.361^*^	0.042	0.050	0.033	0.005	0.000	0.021	0.003	0.000	

*MHQ, Middlesex Hospital Questionnaire; ANX, Anxiety; DEP, Depression; SOM, Somatization; PHOB, Phobia; OBS, Obsession; HY, Hysteria; SF-36, Short Form (36) Health Survey; PF, physical functioning; PR, physical role functioning; BP, bodily pain; GH, general health perceptions, VT, vitality; SR, social role functioning; ER, emotional role functioning; MH, mental health; E, Education; TD, Time spent on dialysis; YT, Years from transplantation; Burden C-19, Burden COVID-19.*

*Sex: Male = 0, Female = 1.*

* *Significance p < 0.05*.

There were no significant correlations between quality of life variables (SF 36) and Burden of COVID-19. Multivariate linear regression analysis did not show significant associations between the predictive variable (Burden COVID-19) and the outcome variables (Anxiety, Phobia, Obsessions, Depression, Hysteria) of the MHQ. However, there was a significant association between the predictive variable “Burden COVID-19” and the “Somatization” outcome variable of the MHQ (Results are shown in Table 3).

**Table 3:** Linear model of predictors ANX, DEP, SOM, PHOB, OBS, HY of “Fear of Covid-19” with 95% bias corrected and accelerated confidence intervals reported in parentheses.

	**B**	**Std. error**	**Beta**	***P***
Constant	8.78	1.29	0.00 (6.12 to 11.45)	0.000
ANX	0.01	0.23	0.01 (−0.47 to 0.49)	0.981
DEP	0.18	0.28	0.15 (−0.39 to 0.74)	0.530
SOM	0.66	0.22	0.62 (0.20 to 1.12)	0.006^*^
PHOB	0.01	0.22	0.01 (−0.44 to 0.46)	0.973
OBS	0.06	0.21	0.06 (−0.37 to 0.50)	0.761
HY	−0.23	0.22	−0.19 (−0.69 to 0.23)	0.318

*ANX, Anxiety; DEP, Depression; SOM, Somatization; PHOB, Phobia; OBS, Obsession; HY, Hysteria.*

* *Significance p < 0.05*.

Paired-sample *t*-test analysis showed no significant change in the mental (*t* = −0.17; df = 23; *p* > 0.05) and physical (t = 0.10; df = 23; *p* > 0.05) health index (MHI and PHI) of SF-36 in recipients before (MHI M = 44.13; PHI M = 44.33) and during (MHI M = 44.58; PHI M = 44.13) the COVID-19 pandemic (see Table 4).

**Table 4:** Paired samples *t*-test mental health index (SF-36) before and during COVID-19 pandemic.

				**95% Confidence interval of the difference**				
	**Mean**	**Std.deviation**	**Std.error mean**	**Lower**	**Upper**	**t**	**df**	**Sig. (2-tailed)**
MHI	−0.46	13.40	2.73	−6.12	5.20	−0.17	23	0.868
PHI	0.21	9.77	1.99	−3.92	4.33	0.10	23	0.918

*MHI, Mental Health Index (SF-36); PHI, Physical Health Index (SF-36)*.

## Discussion

Of the recipients examined, 50% reported in the questionnaire concerning emotional state and psychophysical well-being a change in their perception of their general state of health due to the COVID-19 pandemic. The most reported emotional component change was anxiety (41.4%).

However, this change did not interfere with the overall functioning of the subjects with respect to the previous year. In fact, the MHI and the PHI, evaluated with the SF-36, did not show a significant change when compared with the evaluation carried out a year before the start of the COVID-19 pandemic. Furthermore, the higher the level of education, the better was the perception of mental health as measured by the SF-36.

Regarding Burden of COVID-19, both age and level of education correlated positively, while no correlation with gender emerged. Among the symptoms evaluated with the MHQ, a positive correlation emerged between age, obsessions, level of education and somatizations.

In previous studies, carried out in the period in which there was no pandemic, it was found that as the age of the recipients evaluated increased, anxious and depressive symptoms increased; while the female gender correlated positively with better therapeutic adherence and, the higher the level of education, the better the mental health (16, 21, 22).

The mental health of kidney transplant recipients does not therefore seem to have suffered particular negative consequences due to the COVID-19 pandemic and isolation.

Even social activities were not compromised, which were likely well-compensated by the use of remote technologies.

In fact, it is very interesting to note how, despite the fact that the recent literature has showed an increase in mental health issues due to the pandemic (7–12), our transplant recipients seemed to have managed this period very well (at least in this first phase of evaluation), despite the perceived change and the higher risk they are exposed to due to immunosuppression and the interrupted transplant clinic visits. As stated by many of them during the online care, the transplant experience had probably given them a certain attention to hygiene, to the prevention of possible infections, and the use of masks that they knew well even before COVID-19, avoiding gatherings. This experience helped them to face this pandemic, in many cases better than “healthy” subjects, and to acquire adequate coping strategies. In fact, a recurring phrase of many transplant recipients was: “For me it has not changed that much, I was already used to this!” Being able to carry out psychological and psychiatric examinations online was also said to be very useful, and among other things, many recipients reported that they had benefited from using technology to keep in touch with their closest friends and family.

An interesting fact that deserves to be commented is the significant association between the predictive variable “burden of COVID-19” and the “somatization” outcome variable of the MHQ. These data indicate that a particularly stressful life event such as that relating to the COVID-19 pandemic and the consequent period of isolation, are posed as possible factors precipitating the onset of disease or modulating its course. In our opinion, the psychoneuroendocrine-immunological literature of recent years explains the presence of the “somatic symptom” as an expression of the relationship between stress and inflammatory phenomena, further weakening the immune response with an increase in individual vulnerability to diseases both in a psychological and somatic sense (23). For example depression, characterized by a prolonged inflammatory state with increased concentrations of inflammatory markers, can increase susceptibility to medical illnesses with a tendency to have more somatic symptoms than non-depressed individuals. The presence of somatic symptoms can interfere with treatment, lead to frequent use of health care, reduced quality of life and overall functioning not entirely justified by the condition of organic disease (24). In our opinion Psychotherapeutic and psychoeducational interventions for stress management both in combination with psychiatric drugs and as a first-line approach, can improve lifestyle and self-management, adaptability, quality of life and adherence to treatment in kidney transplant recipients. It is important to consider this aspect, in order to be able to intervene in a preventive and targeted manner to support and monitor any overt manifestations of pathology with psychic expression.

### Limitations

This study has limitations that should be noted when interpreting the main findings. First, it is an observational and cross-sectional study, which precludes causal inferences among the variables analyzed.

Second, a calculation of the sample size is missing and there may be a bias due to the online compilation of the questionnaires: it is not certain whether the subjects completed the questionnaire without the help of family members, despite the instructions provided. Another limitation is the rather short period of investigation: since the COVID-19 pandemic worsened and lasted longer during the second wave, more mental health disturbances may have evolved in these patients. The authors plan to carry out future follow-ups in these recipients as the long-term psychological implications of this population should be further investigated. Further longitudinal studies with large sample sizes need to validate the present findings.

## Conclusions

Our transplant recipients seemed to have managed the COVID-19 pandemic very well (at least in this first phase of evaluation) probably because they were well-compensated by the follow-ups performed online, the interruption of visits to transplant clinics and, because of immunosuppressive therapy that had already accustomed them to paying particular attention to preventive behaviors against infections and to avoiding gatherings.

Kidney transplant recipients have been shown to have favorable attitudes toward the use of remote technology and this can be a stimulus to drive change in the way care and treatment services are provided (25), also given the fear to go to hospital for regular check-ups. This finding is confirmed by the recent study on the impact of COVID-19 in recipients with heart and lung transplantation (26).

As reported in the literature, it would be interesting to maintain this online care remote visit and interview mode to monitor, on a clinical and psychological level, kidney transplant recipients in subsequent follow-ups (12–18 months), to check for any psychopathological disorders and/or changes in their resilience capacity in the Coronavirus emergency (27).

The impact of COVID-19 on the possible development of discomfort/psychopathological aspects in kidney transplant recipients has not yet been clearly established, and there are still few international studies on the quality of life and response to possible psychopathological consequences of the COVID-19 pandemic in patients with organ transplantation.

This era of COVID-19 requires a high degree of responsibility and the multidisciplinary collaboration of all experts in the transplant and mental health fields (28).

## Data Availability Statement

The raw data supporting the conclusions of this article will be made available by the authors, without undue reservation.

## Ethics Statement

The studies involving human participants were reviewed and approved by Ethics Committee Catania 1 University Hospital Policlinico G. Rodolico-San Marco, Catania, Italy. The patients/participants provided their written informed consent to participate in this study.

## Author Contributions

PV and CD: concept/design. MV and MP: data analysis/interpretation. CD and MP: drafting of the study. MV: critical revision of the study. PV: approval of the study. MP and CD: statistics. RG and FP: data collection. All authors contributed to the article and approved the submitted version.

## Conflict of Interest

The authors declare that the research was conducted in the absence of any commercial or financial relationships that could be construed as a potential conflict of interest.
